# The reach-to-grasp movement in infants later diagnosed with autism spectrum disorder: a high-risk sibling cohort study

**DOI:** 10.1186/s11689-018-9259-4

**Published:** 2018-12-27

**Authors:** Lori-Ann R. Sacrey, Lonnie Zwaigenbaum, Susan Bryson, Jessica Brian, Isabel M. Smith

**Affiliations:** 1 Department of Pediatrics, University of Alberta, Edmonton, Alberta Canada; 2 Autism Research Centre, Glenrose Rehabilitation Hospital, (E209) 10230 - 111 Avenue, Edmonton, Alberta T5G 0B7 Canada; 3 Dalhousie University/IWK Health Centre, Halifax, Nova Scotia Canada; 4 Bloorview Research Institute, Toronto, Ontario Canada; 5 University of Toronto, Toronto, Ontario Canada; 6 Autism Research Centre, IWK Health Centre, Halifax, Nova Scotia Canada

**Keywords:** Autism spectrum disorder, Autism, Reaching, Motor, Motor skills, Infant siblings, Baby siblings

## Abstract

**Background:**

Although autism spectrum disorder (ASD) is characterized by impairments in social communication and the presence of repetitive behavior and/or restricted interests, there is evidence that motor impairments may be a contributing factor to the ASD phenotype. The purpose of this study was to examine the motor act of reaching-to-grasp in children at high risk (HR; with an older sibling diagnosed with ASD) and low-risk (LR; no family history of ASD) for ASD.

**Methods:**

Children were compared for differences in reaching-to-grasp based on sibling status and diagnostic outcome. Children were enrolled between 6 and 12 months of age and the reach-to-grasp movement was scored at 6, 9, (where available) 12, 15, 18, 24, and 36 months of age using the qualitative Skilled Reaching Rating Scale to determine the presence of any group-, age-, or sex-related differences in the mechanics of the reach-to-grasp movement using a Mixed Models analysis. At 36 months, all children underwent a gold-standard diagnostic assessment, which resulted in three outcome groups: HR children diagnosed with ASD (HR-ASD; *n* = 10), HR children not diagnosed with ASD (HR-N; *n* = 10), and low-risk children not diagnosed with ASD (LR; *n* = 10).

**Results:**

The group of children who were later diagnosed with ASD (HR-ASD group) showed higher (worse) total scores on the reach-to-grasp movement, as well as higher scores on the components of Orient, Lift, and Pronate compared to children in the LR and HR-N groups.

**Conclusions:**

Our results support the growing literature indicating that children who are later diagnosed with ASD show impaired early motor performance. These results highlight the importance of early surveillance of children who are at elevated risk for ASD, and early initiatives should focus on early signs of the phenotype, including both movement and sensory differences (prodromal signs) prior to the emergence of diagnostic characteristics.

**Electronic supplementary material:**

The online version of this article (10.1186/s11689-018-9259-4) contains supplementary material, which is available to authorized users.

## Background

Autism spectrum disorder (ASD) is characterized by impairments in social communication and the presence of repetitive behavior and/or restricted interests [2]. There is evidence that motor impairments may be a contributing factor to the ASD phenotype. Many individuals with ASD have gross and fine motor deficits [26, 29, 49, 50, 61, 86], including difficulty performing motor gestures [35, 51, 72], impairments in motor control and motor learning [1, 30, 31, 34, 77], and disturbances in the kinematics of reach-to-grasp (i.e., velocity; [47]), as well as the ability to perform a peg-board placement (reaching and manipulation) task [57]. Much of this work was conducted with children or adults who already had a diagnosis of ASD, and the quality of different components of the movement was not compared between groups. Growing evidence indicates that motor impairments appear in the first year of life [4, 43, 80, 87], preceding differences more directly related to ASD diagnostic criteria (reviewed by [88]).

Prospective assessment of younger siblings of children with a diagnosis of ASD (“baby siblings”) who are at heightened risk for also being diagnosed with the disorder (approximately 18%; [58]) provides an opportunity to explore ASD-related behavioral differences early in life. With respect to motor impairment, research using the baby sibling design has compared composite scores between high-risk children who were diagnosed versus not diagnosed with ASD [8, 19, 37, 41] and the attainment of gross or fine motor skills on developmental measures, such as the Mullen Scales of Early Learning [33, 55]. Results of these studies indicate that children who were later diagnosed with ASD had lower (i.e., poorer) overall composite scores on measures of motor skills/development compared to community controls and/or other high-risk children who were not diagnosed with ASD. However, examining composite scores or the absence of specific motor behaviors only provides part of the picture—it is also important to understand whether the mechanics of motor behavior differ based on risk status. The reach-to-grasp movement provides an opportunity for a more detailed examination of motor development, in terms of movement quality and developmental timings for different components of the reach, and is also of functional significance.

The reach-to-grasp movement, in which a hand advances towards an object to grasp with the digits, is a natural act and is displayed in a variety of forms by developing infants and adults. Newborn infants will automatically grasp objects that have been placed in the hand [11, 81], 4-month-old infants begin to reach for distal objects [12, 24, 59], and 12-month-old infants display arm movements and hand grasps that approximate those of adults [22, 67, 79, 85]. The topography of the reach-to-grasp movement has been standardized using healthy young and older adults [66], adults with Huntington’s disease [36], Parkinson’s disease [66, 68], and recovering from stroke [23]. Thus, reaching-to-grasp is a robust movement that can be used to study the development of skilled hand movement and its sensory control, beginning in infancy and continuing across the lifespan. Detailed description of the development of the reach-to-grasp movement beyond 12 months in typically developing infants is still warranted, in addition to potential developmental differences in the movement in children at HR for ASD.

The purpose of this study was to examine whether the reach-to-grasp movement is impaired in children at high risk (HR; with an older sibling diagnosed with ASD) for ASD, relative to controls. Infants were recruited from HR and low-risk (LR; no family history of ASD) families to compare potential differences in reaching-to-grasp based on sibling status and diagnostic outcome. The reach-to-grasp movement was recorded from two ASD assessments, the Autism Observation Scale for Infants (AOSI; ages 6, 9 (where available), 12, and 15 months; [10]) and the Autism Diagnostic Observation Schedule (ADOS; ages 18, 24, and 36 months; [44]). The movement was analyzed using the Skilled Reaching Rating Scale [67] to determine the presence of any group- or age-related differences in the mechanics of the reach-to-grasp movement.

## Method

### Participants

Thirty infants from a longitudinal study of infant siblings of children with ASD (see [10, 89]) participated in the current study. The main study tracked HR infant siblings (each with an older biological sibling diagnosed with ASD) and LR controls (no family history of ASD) to document differences in early development potentially associated with ASD. All infants were enrolled between 6 and 12 months of age and underwent comprehensive assessments of communication, social, cognitive, and motor abilities at ages 6, 9 (where available), 12, 15, 18, 24, and 36 months. Infants in the present study were a subsample of participants from the Autism Research Centre at the Glenrose Rehabilitation Hospital (GRH), Edmonton, Alberta.

Infants were selected at random to comprise three groups of equal size: (1) 10 non-sibling controls (LR-control; 7 boys); (2) 10 HR siblings without an ASD diagnosis (i.e., with an older sibling with ASD but did not receive an ASD diagnosis themselves at 36 months; HR-N; 3 boys); and (3) 10 HR siblings with an ASD diagnosis (i.e., with an older sibling with ASD and also received an ASD diagnosis at 36 months; HR-ASD; 6 boys). The HR siblings were recruited from families following assessment of the older sibling with ASD at the GRH. The diagnosis of the older sibling (or “proband”) was based on evaluation by a multi-disciplinary team and expert clinical review using DSM-IV-TR criteria, supported by a comprehensive developmental history and the Autism Diagnostic Observation Schedule (ADOS; [44]). The LR controls were recruited from the local community on the basis of having no first- or second-degree relatives with an ASD diagnosis. All participants were born at 36 to 42 weeks gestation, had a birth weight greater than 2500 g, and no known genetic or neurological disorders. Table 1 presents detailed participant characteristics. The local institutional review board approved the research protocol and parents provided written informed consent.

**Table 1:** Participant characteristics

	LR controls	HR-N	HR-ASD
Number	10	10	10
Age first visit (mos)	6.94 ± 1.82	7.06 ± 1.29	8.56 ± 2.14
Age diagnostic visit (mos)	36.84 ± 1.13	37.23 ± 1.8	37.66 ± 1.98
Sex	7 boys; 3 girls	3 boys; 7 girls*	6 boys; 4 girls^o^
AOSI total score			
6 months	11.11 ± 4.13	10.38 ± 3.58	14.75 ± 2.50
9 months	6.89 ± 1.83	8.89 ± 3.55	7.22 ± 2.77
12 months	5.00 ± 2.05	7.60 ± 3.41	9.70 ± 5.21*
15 months	4.50 ± 3.72	8.40 ± 4.86	10.0 ± 5.16*
ADOS severity score			
18 months	7.70 ± 5.29	9.20 ± 5.40	14.80 ± 5.90*^o^
24 months	5.50 ± 3.03	6.70 ± 4.90	14.90 ± 6.10*^o^
36 months	4.20 ± 4.94	5.00 ± 3.50	17.30 ± 4.10*^o^
ADI–R total score	6.60 ± 2.76	9.10 ± 2.28	19.80 ± 10.80*^o^
MSEL ELC	123.60 ± 15.71	106.60 ± 11.31	96.44 ± 21.18*
VABS ABC	102.20 ± 7.21	89.88 ± 12.03	75.83 ± 11.94*

Values reported are means + standard deviations. Significant difference from *LR control; ^o^HR-N, *p*s < .0167. Note: reaches were scored at 6 and 9 months (where available) and at 12, 15, 18, 24, and 36 months

*LR* low-risk, *HR-N* high-risk and not diagnosed with ASD, *HR-ASD* high-risk and diagnosed with ASD, *N* total sample, *mos* months, *AOSI* Autism Observation Scale for Infants, *ADOS* Autism Diagnostic Observation Schedule, *ADI* Autism Diagnostic Interview—Revised, *MSEL ELC* Mullen Scales of Early Learning Early Learning Composite ELC, *VABS ABC* Vineland Adaptive Behavior Scales Adaptive Behavior Composite ABC

### Assessments administered

Several assessments were administered to track cognitive and ASD-specific characteristics over time; Table 1 presents a summary of these.

#### Mullen Scales of Early Learning (MSEL; [54])

The MSEL consists of five scales, four of which (Visual Reception, Receptive Language, Expressive Language, and Fine Motor) assess nonverbal, cognitive, and language ability, while the fifth scale measures gross motor development (from 0 to 29 months only). An Early Learning Composite is calculated based on scores from the first four scales for children aged 0–69 months. Inter-rater and test-retest reliability are excellent [54]. The MSEL was administered at 36 months of age.

#### *Vineland Adaptive Behavior Scales I*/*II* (Vineland; [74])

The Vineland is a semi-structured parent interview designed to assess daily living, communication, social, and motor functioning in everyday life, outlined by typical developmental milestones anchored to specific ages. The Vineland has excellent reliability and concurrent validity and is reported to be sensitive to impairments experienced by children with ASD [13, 83]. The Vineland was administered at 36 months of age.

#### Autism Observation Scale for Infants (AOSI; [10])

The AOSI is a semi-structured, observational measure designed to detect and monitor early signs of ASD in infants aged 6 to 18 months. The AOSI uses “presses” to elicit various target behaviors, including visual tracking and attention disengagement, coordination of eye gaze and action, imitation, affective responses, early social-communicative behaviors, and behavioral reactivity. A “press” is either a verbal or non-verbal request with the goal of eliciting behavior in the child. For example, a press for a “social smile” would involve the assessor looking at the child and smiling, with the goal of eliciting a reciprocal smile from the child. The AOSI was administered at 6, 9, 12, and 15 months of age.

#### Autism Diagnostic Observation Schedule (ADOS; [44])

The ADOS includes standardized activities and presses to elicit communication, social interaction, imaginative use of play materials, and repetitive behavior. Inter-rater reliability for the ADOS is excellent [44]. The scoring algorithm is organized into two domains, Social Affect (including Communication and Social items), and Restricted Repetitive Behaviors [28]. The ADOS consists of four modules, each appropriate for individuals of differing language levels (module 1 = minimal or no language, module 2 = regular use of non-echoed three-word phrases, module 3 = child with fluent language, and module 4 = adolescent or adult with fluent language), the first three of which were used to assess participants in this study. To compare across modules (and thus, across language levels), we used the ADOS severity metric [27]. The ADOS was administered at 18, 24, and 36 months of age.

#### *Autism Diagnostic Interview—Revised* (ADI-R; [45])

The ADI-R is an investigator-directed interview that elicits information regarding social development, verbal and non-verbal communication skills, and the presence of repetitive, stereotyped interests, and behavior required to make an ICD-10 or DSM-IV-TR diagnosis of ASD. The questions are designed to distinguish qualitative impairments from developmental delays. The ADI-R discriminates well between ASD and other forms of developmental disability, and inter-rater reliability is excellent [45]. The ADI-R was administered to parents when the children were at 36 months of age.

### Diagnostic procedure

At 3 years of age, each participant underwent an independent diagnostic evaluation, conducted by an expert clinician blind to assessments from previous study visits. ASD diagnoses were assigned using DSM-IV-TR criteria, based on the best judgment of the clinician (developmental pediatrician with over 10 years of diagnostic experience), considering information from all concurrent assessments. Because the majority of our sample was diagnosed prior to 2013 (when DSM-5 was published), we continued to use DSM-IV-TR criteria for diagnoses to remain consistent.

### Assessments scored for reach-to-grasp

Reach-to-grasp was coded from the video recordings of the AOSI and the ADOS by an individual who was not involved in the assessments and was blind to sibling status and diagnostic outcomes.

#### Autism Observation Scale for Infants (AOSI; [10])

Reach-to-grasp measures were coded from the AOSI at 6, 9 (where available), 12, and 15 months of age using the two *Free Play* sections. These together are approximately 10 min, with the first section occurring at the beginning and the second at the end of the AOSI. The two sections are separated by two tasks (peek-a-boo and imitation), lasting approximately 5 min. During the free play sessions, the child sits at a table across from the examiner, either seated in her/his parent’s lap or alone in a posture supportive chair, with hands and arms free to grasp and manipulate objects. Briefly, the examiner places a variety of graspable items on the table in front of the infant and encourages the child to reach for and grasp the items. The *Free Play* sections of the AOSI were chosen because (1) the child is encouraged to pick up small items (blocks, rings) for manipulation; (2) interruptions by the examiner are minimal; and (3) the two sections constitute the majority of the AOSI, allowing ample opportunity to sample grasps sufficiently (see below). Infants reached for the same set of toys at each age.

#### Autism Diagnostic Observation Schedule (ADOS; [44])

Reach-to-grasp measures were coded from the ADOS at 18, 24, and 36 months of age using the *Birthday Party* routine. The *Birthday Party* routine occurs at the end of the ADOS (followed only by “snack”) and is approximately 10 min. During this routine, the child sits at a table across from the examiner with hands and arms free to grasp and manipulate objects. Briefly, during the *Birthday Party*, the examiner “makes a cake” from play-dough and encourages the child to grasp and place candles in the cake. After singing “Happy Birthday,” the child is encouraged to feed cake and offer drinks to the birthday baby (i.e., the examiner says, “baby’s hungry” with the expectation that the child will “feed” the baby). The ADOS *Birthday Party* routine was chosen for scoring because (1) the child is encouraged to pick up small items (candles, fork, knife) for manipulation; (2) this is one of the longer manipulation sections of the ADOS, providing opportunities to gather grasps; and (3) the *Birthday Party* routine is included in the two ADOS modules used to assess 18- to 36-month-olds in this study.

### Reaches sampled

The first five successful reach-to-grasp movements were sampled for each infant at each time point. To be included as a sampled reach, the infant had to make an overt eye movement towards the target, reach his/her hand towards the target, grasp it, lift the target from the substrate, and make an overt eye movement to disengage from the target. Some infants did not perform five successful reaches in a given session (e.g., did not lift or manipulate an object). Table 2 displays the total number of reaches scored at each time-point, for each infant.

**Table 2:** Total number of reaches scored

Age	Assessment scored	LR control	HR-N	HR-ASD	Total
6 months	AOSI	40	40	20	100
9 months	AOSI	40	40	45	125
12 months	AOSI	45	50	50	145
15 months	AOSI	45	45	45	135
18 months	ADOS	42	50	50	142
24 months	ADOS	47	46	43	136
36 months	ADOS	37	49	38	124
Total		296	320	291	907

*LR* low-risk, *HR-N* high-risk and not diagnosed with ASDASD, *HR-ASD* high-risk and diagnosed with ASD, *AOSI* Autism Observation Scale for Infants, *ADOS* Autism Diagnostic Observation Schedule. Note: reaches were scored at 6 and 9 months (where available) and at 12, 15, 18, 24, and 36 months

### Reach-to-grasp coding

The Skilled Reaching Rating Scale is based on a description of reaching derived from a conceptual framework using Eshkol-Wachman Movement Notation (EWMN; [18, 76, 77]). The topography of the reach-to-grasp movement has been standardized using healthy young and older adults [66], adults with Huntington’s disease [36], Parkinson’s disease [66, 68], and stroke [23]. Sacrey et al. [67] adapted the scoring system to assess its utility in infant development by comparing the absence/presence of subcomponents of the movement compared to the typical adult construct. The results showed that the reach-to-grasp movement undergoes dramatic changes from 6 to 12 months of age, from an uncoordinated, immature movement at 6 months to an act that closely resemble the adult reach-to-grasp by 12 months. This study extends this research by examining the development of the reach-to-grasp movement beyond 12 months in typically developing infants, but also aims to explore potential developmental differences in the movement in children at HR for ASD.

The movement is divided into five components:

1. *Orient*: participant moves the head and eyes in order to fixate the target visually prior to reach onset and visually disengage from the target at grasp.
2. *Lift*: hand is lifted and supinated towards the midline of the body as the digits close and semi-flex
3. *Advance*: hand is carried towards the target and stops above the target
4. *Pronation*: hand pronates over target item and digits shape to target size
5. *Grasp*: target is grasped using a pincer grasp (thumb and index) or appropriate
  grasp for object size

The five components are further divided into 14 subcomponents (for a complete description, see Table 3). The developmental trajectory of reaching was scored based on infant performance relative to healthy adult performance. For rating, a score of “0” was given if the movement was present and resembled the adult construct, a score of “0.5” was given if the movement was present, but differed from the adult construct, and a score of “1” was given if the movement was absent. Thus, lower scores reflect better movement quality relative to that of adults [84]. The first study to standardize reaching-to-grasp for humans reported no significant difference between scores from four different raters [84]. The blind coder established reliability on a standard set of infant reach-to-grasp reliability videos (a total of 168 reaches). Inter-rater reliability was assessed using Pearson’s *r*, resulting in *r* = 0.916, *p* < 0.002, suggesting high reliability. For a detailed description of the video recording procedure, EWMN, and movement onset and offset definitions, see Additional file 1.

**Table 3:** Skilled Reaching Rating Scale

Phase	Component	Sub-component	Description
Orient	Orient	A	Eyes locate target prior to movement of head/reach
		B	Eyes disengage target at tactile contact
Transport	Lift	A	Initial hand lift due to flexion of the elbow
		B	Digits semi-flex
		C	Hand supinates approximately 30 degrees
		D	Tips of digits are brought towards the midline of the body
	Advance	A	Hand takes shortest path to target
		B	Hand stops directly above the target
		C	Trunk leans to the side opposite the reach
	Pronation	A	Digits open and extend over the target
		B	Knuckle on reaching hand form horizontal line
		C	Elbow opens to full arm length as participant reaches
Grasp	Grasp	A	Thumb and index finger/proper grasp for target size/shape
		B	Wrist extends to lift target from platform

### Procedure

Video recordings of AOSI administration at 6, 9 (where available), 12, and 15 months of age and of the ADOS at 18, 24, and 36 months of age were collected and given to the coder, who was blind to both sibling status and diagnostic outcome until all reaches were scored. The children were scored for reach-to-grasp measures at each time point in chronological order; that is, records were scored for one child at a time until all were scored, and each infant’s videos were scored at 6 months, then 9, through to 36 months. The scores from each time point were stored in a binder and were not referenced during the following scoring in an attempt to minimize the influence of the previous scoring on subsequent time points. After all records were scored at each of the seven time points, participants were placed into groups based on sibling status and 36-month diagnostic outcomes.

### Statistical analysis

The first five successful reaches per infant per time point were included in the analysis. Given instances in which an infant made less than five successful reach and grasp movements, particularly for the HR-ASD group at 6 months, scores of each component of the reach for each infant were transformed into averages across trials. The Statistical Package for the Social Sciences (SPSS) v. 21 was used to run a Linear Mixed Model analysis with group (LR control, HR-N, HR-ASD), age (6, 9, 12, 15, 18, 24, and 36 months), and sex (boy, girl; due to the disproportionate number of boys in the HR-ASD and LR control groups) as the independent variables and reach-to-grasp components as the within-subjects measures. Benjamini and Hochberg [7] corrections were applied to all post hoc comparisons. This method controls the expected proportion of rejected null hypotheses that are false. The *p* values are ordered smallest to largest. The alpha level for each test is then set at $\frac{k\ast \alpha }{m}$ with *k* corresponding to the *p* value’s rank (e.g., lowest *p* value = 1) and *m* corresponding to the number of comparisons, which in this case was 21. The comparisons stop once one of the *t* tests is rejected and a resultant *q* value is generated. The *q* value is then used as the new “alpha” for post hoc comparisons. Effect sizes were calculated using Cohen’s *d*, with 0.2–0.49 = small effect, 0.5–0.79 = medium effect, and 0.8+ = large effect [14].

## Results

### Participant characteristics

Groups were compared on cognitive/developmental (MSEL), adaptive behavior (VABS), and ASD symptom (AOSI, ADOS, and ADI-R) scores. As shown in Table 1, children who were diagnosed with ASD differed from the LR controls and HR-N groups on these measures. Ages at first visit and diagnostic visit were compared. As shown in Table 1, groups did not differ in age at initial assessment (*F*(2,27) = 2.43, *p* > 0.05) or diagnostic assessment (*F*(2,27) = 0.59, *p* > 0.05). More girls were in the HR-N group compared to the LR control group and the HR-ASD groups (*χ*^*2*^ = 31.08, *p* < .001; *χ*^*2*^ = 25.47.08, *p* < .001, respectively), who did not differ (*χ*^*2*^ = .50, *p* = .49).

### Reach-to-grasp movement

The group means, standard deviations, and 95% confidence intervals for the components, sub-components, and total reach-to-grasp scores are provided in Table 4.

**Table 4:** Group means, standard deviations, and 95% CI for the reach-to-grasp measures

Measure	LR	HR-N	HR-ASD
Orient	.65 ± .26 (.56–.75)	.65 ± .26 (.56–.75)	.81 ± .26 (.72–.91)
Engage	.20 ± .14 (.15–.25)	.24 ± .15 (.19–.29)	.20 ± .13 (.16–.25)
Disengage	.41 ± .17 (.35–.47)	.51 ± .18 (.44–.57)	.51 ± .16 (.45–.57)
LIFT	1.03 ± .50 (1.12–1.48)	1.39 ± .54 (1.19–1.58)	1.73 ± .48 (1.56–1.90)
Elbow flex	.10 ± .11 (.06–.13)	.13 ± .12 (.09–.17)	.18 ± .10 (.14–.21)
Digits flex	.40 ± .18 (.34–.47)	.44 ± .19 (.37–.51)	.53 ± .17 (.47–.59)
Supinate	.36 ± .17 (.30–.42)	.34 ± .17 (.27–.41)	.46 ± .17 (.40–.52)
Tips to midline	.44 ± .18 (.38–.51)	.48 ± .19 (.41–.55)	.57 ± .17 (.51–.63)
Advance	.45 ± .21 (.38–.53)	.49 ± .22 (.41–.57)	.56 ± .20 (.49–.64)
Hand path	.25 ± .13 (.20–.29)	.28 ± .14 (.23–.33)	.28 ± .13 (.24–.33)
Hand to target	.20 ± .12 (.15–.24)	.21 ± .12 (.16–.25)	.26 ± .11 (.22–.30)
Trunk lean	.01 ± .05 (.00–.03)	.00 ± .05 (.00–.02)	.02 ± .05 (.01–.04)
Pronation	.82 ± .32 (.70–.93)	.91 ± .35 (.78–1.03)	1.10 ± .31 (.99–1.21)
Digits open	.44 ± .20 (.37–.52)	.51 ± .22 (.43–.59)	.59 ± .19 (.52–.66)
Knuckle	.37 ± .16 (.32–.43)	.40 ± .17 (.33–.46)	.51 ± .15 (.45–.56)
Elbow extend	.001 ± .02 (.0–.01)	.01 ± .01 (.00–.01)	.00 ± .01 (.00–.01)
Grasp	.93 ± .23 (.85–1.01)	.89 ± .25 (.81–.98)	.96 ± .22 (.88–1.04)
Appropriate grasp	.23 ± .14 (.18–.28)	.24 ± .15 (.18–.29)	.25 ± .13 (.20–.30)
Wrist extend	.70 ± .19 (.63–.76)	.66 ± .20 (.59–.73)	.71 ± .18 (.65–.76)
Reach-to-grasp	4.15 ± 1.04 (3.78–4.53)	4.52 ± 1.12 (4.11–4.92)	5.17 ± 0.99 (4.81–5.53)

#### Total score

**Fig. 1:**
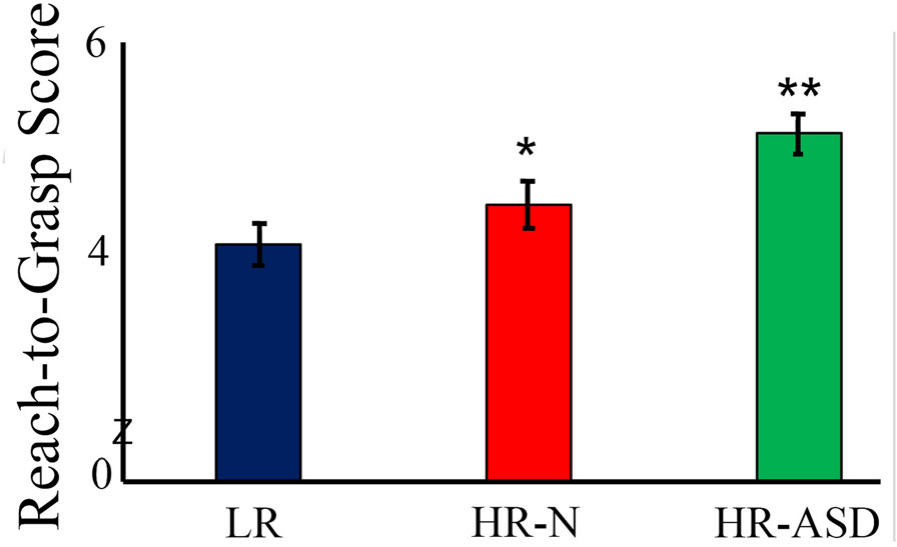
Main effect for reach-to-grasp score. LR, low-risk controls; HR-N, high-risk siblings without autism spectrum disorder; HR-ASD, high-risk siblings with autism spectrum disorder. *p < .05; **p <. 01

As displayed in Fig. 1, significant effects were seen for group (*F*(2,144) = 7.66, *p* = .001), age (*F*(6, 144 = 72.72, *p* < .001), and sex (*F*(1, 144) = 5.41, *p* = .02), but no significant interactions emerged (*p*s > .05). G*Power was used to calculate estimated power for the overall group effect, resulting in a value of 0.74. Post hoc analyses showed that children in the HR-ASD group had higher (worse) reach-to-grasp scores compared to children in the HR-N and LR groups (*q*s < .033; *d* = .48, *d =* .74, respectively), who did not differ (*d* = .26). Post hoc analyses on the age effect revealed improvements in the overall reach-to-grasp score for all time point comparisons at 6, 9, and 12 months, suggesting overall improvement in reaching in the first year of life, which plateaus for each successive time point (*q*s < .038). The sex effect showed that boys (mean = 4.35) had significantly lower (better) overall reach-to-grasp scores than girls (mean = 4.87; *p* < .02; *d* = 1.96).

#### Component analyses

Group comparisons were made on components to determine if different aspects of the reach-to-grasp movement differed between children with ASD and the other two groups. Overall, children with ASD showed differences in the Orient, Lift, and Pronate components. These results are described below.

*Orient*. Significant effects were seen for group (*F*(2,144) = 4.08, *p* = .019) and age (*F*(6, 144) = 22.69, *p* < .001), but not sex (*F*(1, 144) = 1.97, *p* = .16), nor significant interactions (*p*s > .05). Post hoc analyses on the group effect showed that children in the LR group had a lower (better) Orient score compared to children in the HR-N and HR-ASD groups (*q*s < .033; *d* = .51; *d* = .47), who did not differ (*d* = .05). Post hoc analyses on the age effect showed that improvements in Orient began between 9 and 12 months and continued to age 3 (*q*s < .043).

*Lift*. Significant effects were obtained for group (*F*(2, 144) = 6.51, *p* = .002) and age (*F*(6, 144) = 46.60, *p* < .001), but not sex (*F*(1, 144) = .3.66, *p* = .06) or interaction effects (*p*s > .05). Post hoc analyses on the group effect showed that children in the HR-ASD group had a higher Lift score than the LR-control group (*q* < .017). Children in the HR-N group did not differ from the other two groups. Post hoc analyses on the age effect showed that Lift improved for all time-point comparisons at 6, 9, and 12 months and plateaued for each successive time point (*q*s < .036).

*Advance*. A significant effect was seen for age (*F*(6, 144) = 67.27, *p* < .001), but not group (*F*(2, 144) = 2.41, *p* = .09), sex (*F*(1, 144) = .1.25, *p* = .27), or interaction effects (*p*s > .05). Post hoc analyses on the age effect showed that Advance improved for all time-point comparisons at 6, 9, and 12 months and plateaued for each successive time point (*q*s < .036).

*Pronate*. Significant effects were obtained for group (*F*(2, 144) = 6.17, *p* = .003), age (*F*(6, 144) = 33.14, *p* < .001), and sex (*F*(1, 144) = 10.77, *p* = .001), with no significant interactions (*p*s > .05). Post hoc analyses on the group effect showed that children in the HR-ASD group had a higher (worse) Pronate score compared to children in the LR and HR-N groups (*q*s < .033; *d* = .66; *d* = .41, respectively), who did not differ (*d* = .21). Post hoc analyses on the age effect showed that Pronate improved for all time point comparisons at 6, 9, and 12 months and plateaued for each successive time point thereafter (*q*s < .036). The results of the sex effect showed that boys (mean = .83) had lower (better) Pronate scores than girls (mean = 1.05; *p* = .001; *d* = 2.75).

*Grasp*. A significant effect was seen for age (*F*(6, 144) = 18.59, *p* < .001), but not group (*F*(2, 144) = .61, *p* = .55), sex (*F*(1, 144) = 1.39, *p* = .24), or interaction effects (*p*s > .05). Post hoc analyses on the age effect showed that Grasp improvements began between 9 and 12 months and plateaued by age 15 months (*q*s < .029).

#### Sub-component analysis

The results of the sub-component analyses are summarized in Table 5. Briefly, eight sub-components showed group differences: disengage of Orient, elbow flex, digits flex, supinate, and tips to midline of Lift, and digits open, knuckle, and elbow of Pronation.

**Table 5:** Sub-component analysis results

Component	Sub-component	Group		Age		Sex		Interaction effects	Post hoc group^O^
		*F*	*p*	*F*	*p*	*F*	*p*		
Orient	A. Engage	.63	.53	5.33	.001*	2.27	.13	No	NA
	B. Disengage	3.38	.04*	18.65	.001*	1.12	.29	No	a < b, c
Lift	A. Elbow flex	4.61	.01*	44.56	.001*	.62	.43	No	a < c
	B. Digits flex	4.33	.02*	23.79	.001*	2.57	.11	No	a < c
	C. Supinate	3.77	.03*	34.01	.001*	6.53	.01*^	No	b < c
	D. Tips to midline	3.91	.02*	24.12	.001*	2.88	.09	No	a < c
Advance	A. Hand path	.86	.43	37.47	.001*	1.76	.19	No	NA
	B. Hand to target	2.53	.08	56.89	.001*	.001	.98	Yes^1^	NA
	C. Trunk lean	1.58	.21	1.04	.40	1.41	.24	No	NA
Pronation	A. Digits open	4.02	.02*	15.87	.001*	8.71	.004*^	No	a < c
	B. Knuckle	6.57	.002*	43.81	.001*	9.24	.003*^	No	c > a, b
	C. Elbow extend	3.39	.04*	4.17	.001*	5.65	.019*^	Yes^1234^	b < c
Grasp	A. Appropriate grasp	.13	.88	13.03	.001*	.11	.75	Yes^1^	NA
	B. Wrist extenda	.60	.55	10.51	.001*	2.95	.09	No	NA

*a* LR control; *b* HR-N, *c* HR-ASD, *NA* not applicable; *Significantly different at *p* < .05, ^O^Benjamini and Hochberg corrected *p* value

^1^Age × sex interaction

^2^Group × sex interaction

^3^Age × sex interaction

^4^Group × age × sex interaction

^^^Boys < girls

## Discussion

The present study examined mechanical differences between the reach-to-grasp movements of infants and toddlers who were at high and low risk for ASD. Children performed the movement at 6, 9 (where available), 12, 15, 18, 24, and 36 months of age and the movement was scored using the qualitative Skilled Reaching Rating Scale [84]. Children who were later diagnosed with ASD showed higher (worse) total scores on the reach-to-grasp movement, as well as higher scores on the components of Orient, Lift, and Pronate compared to children in the LR and HR-N groups. These results suggest that such movement mechanics are relevant to monitoring motor development in children at risk for or diagnosed with ASD.

Our study of the reach-to-grasp movement is, to our knowledge, the first to examine the quality of various components of the movement prior to the diagnosis of ASD. Much of the research examining reach-to-grasp movements in ASD has either been completed with children or adults who already have a diagnosis or employed kinematics (velocity/acceleration) to study potential group differences [16, 25, 47, 75, 82]. Nevertheless, our results are consistent with previous reports, indicating these movements are less well-coordinated in children with ASD compared to age-matched typically developing controls [20, 31]. The emerging reach-to-grasp movements of children in our study who were later diagnosed with ASD appeared less well-coordinated than the more adult-like movements of LR controls and also differed from those of HR children who were not diagnosed with ASD by age 3.

Previous research examining motor impairments, specifically reaching and grasping, which identified group differences often had only LR comparison groups [20, 25, 47, 75]. Additional comparison groups were children with various developmental disabilities (DD; [16, 31]) or developmental coordination disorder (DCD; [82]). Results from these studies are mixed, some finding differences between ASD and DD or DCD, and others not. This is problematic, as other control groups (e.g., developmental delay, HR-non-ASD siblings, in addition to LR controls) are necessary to separate putative ASD-specific impairments from more general delays [71].

Children with ASD show impairments in the disengage subcomponent of orienting. This finding is consistent with the literature, noting the presence of “sticky attention” in children who are diagnosed, or HR infant siblings who are later diagnosed, with ASD (reviewed in [64]). Delays in disengagement may affect joint attention, spontaneous gaze to faces, orienting to name, and making eye contact, which all involve orienting visual attention to biologically relevant information in the environment [38, 39, 58, 89]. Furthermore, sticky attention may combine with motor impairments to further impact an individual’s ability to participate in collaborative activities (i.e., delays in responding to visual information in addition to motor deficits). Our finding here, combined with previous research suggesting impaired disengagement of attention is apparent at 12 months of age [9, 65], can distinguish between HR siblings who later receive an ASD diagnosis [9, 17, 65], and can distinguish between ASD and Down syndrome [39], suggests that “sticky attention” can be a marker in the early identification of children at risk for ASD.

Neuroimaging research has identified several cortical and subcortical abnormalities in areas known to be involved in motor behavior [5, 6, 15, 48, 52, 56, 62]. For example, cerebellar abnormalities have been identified in individuals with ASD (reviewed in [21]). The cerebellum, a key structure required to form internal models of motor actions, shares reciprocal connections with motor cortices to carry out online corrections during movement execution [53, 78], and these abnormalities likely contribute to the impaired online movement correction seen in ASD. Abnormal connectivity between adjacent primary sensory and motor cortices has also been recorded in ASD [70, 75] and may also contribute to the impairments seen during the online control of movement [46]. Of note here, reduced inter-connectivity between distal areas of the motor system, such as visual and motor regions subserving action, may lead to impaired motor planning and execution in individuals with ASD [70, 75]. Future research could link behavioral and neuroimaging data to determine whether impairments in the reach-to-grasp movement are associated with specific brain areas/abnormalities.

Motor ability is linked to cognitive outcomes in individuals with ASD [3, 60, 73]. This relationship may be mediated by the effects of motor impairments on opportunities for learning through everyday experience. As an illustration, consider the motor milestone of independent sitting. If a child is delayed in achieving independent sitting and therefore spends a large portion of his/her time on the tummy, this reduces opportunities to use the hands to engage in reaching and grasping for objects, showing objects to caregivers, and even requesting objects. As a consequence, the onset of these “social” behaviors may in turn be delayed. This is supported by a 2011 study from Libertus and Needham, who found that typically developing babies who engage in active reaching movements also show spontaneous orienting to faces. This is in contrast to babies who watch others play with objects, having not yet achieved active reaching skills, and show less spontaneous orienting to faces. Like sitting, reaching-to-grasp is typically acquired in the first year [67] and disruptions may have further implications for more sophisticated object exploration (e.g., [63]). Reaching provides infants with the opportunity to play with toys spontaneously, and the ability to grasp toys and refine the grasp to adjust to the shape or features of the toy allows the child to maintain control while engaging in exploratory behavior. As such, difficulties with reaching and grasping may constrain these interactions and limit the infant’s ability to explore objects effectively [33]. Furthermore, infants impaired in their ability to grasp and manipulate objects often also show delays in speech onset and development (reviewed in [32]), suggesting that the ability to reach for and grasp objects may have cascading effects on other emerging skills.

How do motor impairments relate to social-communicative impairments? A typically developing child can use a full movement repertoire to engage in social interactions. Yet, many children with ASD have impaired motor behavior, detectable as early as 3 months of age [8]. As proposed by Leary and Hill [40], motor ability might have a significant impact on the core characteristics of ASD. Specifically, when a person is unable to respond to another’s action in a timely fashion, he/she will miss the positive reinforcement associated with interpersonal interactions. As well, the child’s suboptimal response may negatively affect their relationship with that person (especially with a peer), reducing the likelihood of subsequent positive interactions. Such consequences may be overt during an interaction centered on motor activities (e.g., team sports such as baseball, which centers on cooperative motor tasks such as catching and throwing a ball), but may also occur during a broader range of social interactions that involve responding to another’s actions. Thus, experiences throughout development may be drastically altered if, at an early age, a child is unable to remain involved in social interaction and, as a result, may withdraw from social activities [40]. This “motor cognition” perspective does not imply that social-communicative impairments directly result from motor impairments, but rather that impaired movements may interfere with opportunities for positive social experiences and thus social learning. Conversely, reduced social interaction opportunities may also contribute to poor action understanding. Thus, the relationship between social and motor competencies/impairments may be reciprocal in ASD, a hypothesis that remains to be explored in future longitudinal research.

Although our results add to the recent literature of motor impairments in children with ASD, it is not without limitations. First, the number of children in the HR-ASD group is low at the earliest age examined (6 months), due to later enrollment in the study, reducing the number of codable reaches. Nevertheless, when we directly compared results inclusive of all data versus data from 12 to 36 months, the pattern of significance was identical, suggesting the overall group results are quite robust (see Additional file 1: Table S1). Second, we acknowledge that the data presented in this paper are based on a relatively small sample (10 participants per group) coded using labor-intensive frame-by-frame analyses. Our smaller sample is similar to that of other papers analyzing reaching movements in ASD (e.g., [47, 57]); yet, those papers only tested their participants at one time point whereas our participants performed reaching movements over multiple time points between 6 (where available) and 36 months of age. Importantly, a large number of reaches were scored (approximately 300 per group) and the power analyses indicated that our study could detect small-to-medium effects as statistically significant because of multiple sampling per participant at each assessment and over time.

## Conclusion

Our results support the growing literature indicating that children who are later diagnosed with ASD show impaired early motor performance. Results such as these highlight the importance of early surveillance of children who are at elevated risk for the disorder. These initiatives should focus on early signs of the phenotype, including both movement and sensory responsivity/interests (prodromal signs) prior to the emergence of diagnostic characteristics (social communication and repetitive behaviors/restricted interests). Reports of parental concerns and early screening instruments note caregivers’ ability to identify prodromal signs [42, 69], and thus may be a preferred source of information for early surveillance efforts.

## Additional file

Additional file 1: **Table S1.** Comparison of Main Effects for including all reaches completed between 6 and 36 months versus reaches completed only between 12 and 36 months. (DOCX 22 kb)

## Abbreviations

ADI-R: Autism Diagnostic Interview—Revised
ADOS: Autism Diagnostic Observation Schedule
AOSI: Autism Observation Scale for Infants
ASD: Autism spectrum disorder
DCD: Developmental coordination disorder
DD: Developmental delay
EWMN: Eshkol-Wachman movement notation
HR: High-risk
HR-ASD: High-risk sibling with autism spectrum disorder
HR-N: High-risk sibling without autism spectrum disorder
LR: Low-risk
MSEL: Mullen Scales of Early Learning
SPSS: Statistical Package for the Social Sciences

## References

[1] Adrien JL, Lenoir P, Martineau J, Perrot A, Hameury L, Larmande C (1993). Blind ratings of early symptoms of autism based upon family home movies. J Am Acad Child Adol Psych.

[2] American Psychiatric Association. Diagnostic and statistical manual (DSM-5). 5th ed. Washington: American Psychiatric Association; 2013.

[3] Bar-Haim Y, Bart O (2006). Motor function and social participantion in kindergarten children. Soc Dev.

[4] Baranek GT (1999). Autism during infancy: a retrospective video analysis of sensory-motor and social behaviours at 9-12 months of age. J Autism Dev Dis..

[5] Bauman ML, Kemper TL (2005). Neuroanatomical observations of the brain in autism: a review and future directions. Int J Dev Neurosci.

[6] Bauman ML, Kemper TL (1994). Neuroanatomical observations of the brain in autism.

[7] Benjamini Y, Hochberg Y (1995). Controlling the false discovery rate: a practical and powerful approach to multiple testing. J Royal Stat Soc Series B.

[8] Bhat AN, Galloway JC, Landa RJ (2012). Relation between early motor delay and later communication delay in infants at risk for autism. Infant Behav Dev.

[9] Bryson SE, Garon N, McMullen T, Brian J, Zwaigenbaum L, Armstrong V (2017). Impaired disengagement of attention and its relationship to emotional distress in infants at high-risk for autistic spectrum disorder. J Clin Exp Neuropsych.

[10] Bryson SE, Zwaigenbaum L, McDermott C, Rombough V, Brian J (2008). The autism observation scale for infants: scale development and reliability data. J Autism Dev Dis.

[11] Butterworth G, Harris M (1994). Principles of developmental psychology.

[12] Butterworth G, Hopkins B (1988). Hand-mouth coordination in the new-born baby. Br J Dev Psychol.

[13] Carter AS, Volkmar FR, Sparrow SS, Wang JJ, Lord C, Dawson G (1998). The Vineland Adaptive Behavior Scales: supplementary norms for individuals with autism. J Autism Dev Dis.

[14] Cohen J (1988). Statistical power analyses for the behavioral sciences (2nd Ed).

[15] Courchesne E (2002). Abnormal early brain development in autism. Mol Psychiatry.

[16] David FJ, Baranek GT, Wiesen C, Miao AF, Thorpe DE (2012). Coordination of precision grip in 2-6 years-old children with autism spectrum disorder compared to children developing typically and children with developmental disabilities. Front Integ Neurosci.

[17] Elsabbagh M, Fernandes J, Webb SJ, Dawson G, Charman T, Johnson MH (2013). Disengagement of visual attention in infancy is associated with emerging autism in toddlerhood. Biol Psych.

[18] Eshkol N, Wachman A (1958). Movement notation.

[19] Estes A, Zwaigenbaum L, Gu H, John TS, Paterson S, Elison JT (2015). Behavioral, cognitive, and adaptive development in infants with autism spectrum disorder in the first 2 years of life. J Neurodev Dis.

[20] Fabbri-Destro M, Cattaneo L, Boria S, Rizzolatti G (2009). Planning actions in autism. Exp Brain Res.

[21] Fatemi SH, Aldinger KA, Ashwood P, Bauman ML, Blaha CD, Blatt GJ (2012). Consensus paper: pathological role of the cerebellum in autism. Cerebellum.

[22] Fiorentino MR (1981). A basis for sensorimotor development, normal and abnormal: the influence of primitive, postural reflexes on the development and distribution of tone.

[23] Foroud A, Whishaw IQ (2010). Reaching-to-eat in humans post-stroke: fluctuating components within a constant pattern. Behav Neurosci.

[24] Foroud A, Whishaw IQ (2012). The consummatory origins of visually guided reaching in human infants: a dynamic integration of whole-body and upper-limb movements. Behav Brain Res.

[25] Forti S, Valli A, Perego P, Nobile M, Crippa A, Molteni M (2011). Motor planning and control in autism. A kinematic analysis of preschool children. Res Autism Spect Dis.

[26] Fournier KA, Hass CJ, Naik SK, Lodha N, Cauraugh JH (2010). Motor coordination in autism spectrum disorders: a synthesis and meta-analysis. J Autism Dev Dis.

[27] Gotham K, Pickles A, Lord C (2009). Standardizing ADOS severity scores for a measure of severity in autism spectrum disorders. J Autism Dev Dis.

[28] Gotham K, Risi S, Pickles A, Lord C (2007). The autism diagnostic observation schedule: revised algorithms for improved diagnostic validity. J Autism Dev Dis.

[29] Greenspan S, Wieder S (1997). Developmental patterns and outcomes in infants and hildren with disorders in relating and communicating: a charting review of 200 cases of children with autistic spectrum diagnoses. J Autism Dev Dis.

[30] Haswell CC, Izawa J, Dowell LR, Mostofsky SH, Shadmehr R (2009). Representations of internal models of action in the autistic brain. Nature Neurosci.

[31] Hughes C (1996). Brief report: planning problems in autism at the level of motor control. J Autism Dev Dis..

[32] Iverson JM (2010). Developing language in a developing body: the relationship between motor development and language development. J Child Lang.

[33] Iverson JM, Shic F, Wall CA, Charwarska K, Curtin S, Estes A, et al. Early motor abilities in infants at heightened vs. low risk for ASD: a Baby Siblings Research Consortium (BSRC) study. J Abn Psych. 2018; Accepted. 10.1037/abn0000390 PMC6338079 30628809

[34] Jansiewicz EM, Goldberg MC, Newschaffer CJ, Denckla MB, Landa R, Mostofsky SH (2006). Motor signs distinguish children with high functioning autism and Asperger’s syndrome from controls. J Autism Dev Dis.

[35] Jones V, Prior M (1985). Motor imitation abilities and neurological signs in autistic children. J Autism Dev Dis.

[36] Klein A, Sacrey LR, Dunnett SB, Whishaw IQ, Nikkhah G (2011). Proximal movements compensate for distal forelimb movement impairments in a reach-to-eat task in Huntington’s disease: new insights into motor impairments in a real-world skill. Neurobiol Dis.

[37] Landa R, Garrett-Meyer E (2006). Development in infants with autism spectrum disorder: a prospective study. J Child Psychol Psych.

[38] Landa R, Holman KC, Garret-Mayer E (2017). Social and communication development in toddlers with early and later diagnosis of autism spectrum disorders. Arch Gen Psych.

[39] Landry R, Bryson SE (2004). Impaired disengagement of attention in young children with autism. J Child Psychol Psych.

[40] Leary MR, Hill DA (1996). Moving on: autism and movement disturbance. Ment Retard.

[41] Leonard HC, Elsabbagh M, Hill EL (2014). BASIS team. Early and persistent motor difficulties in infants at-risk of developing autism spectrum disorder: a prospective study. Eur J Dev Psychol.

[42] Libertus K, Landa RJ (2013). The early motor questionnaire (EMQ): a parent report measure of early motor development. Infant Behav Dev.

[43] Libertus K, Sheperd KA, Ross SW, Landa RJ (2014). Limited fine motor and grasping skills in 6-month-old infants at high risk for autism. Child Dev.

[44] Lord C, Risi S, Lambrecht L, Cook EH, Leventhal BL, DiLavore PC (2000). The autism diagnostic observation schedule-generic: a standard measure of social and communication deficits associated with the spectrum of autism. J Autism Dev Dis.

[45] Lord C, Rutter M, LeCouteur A (1994). Autism diagnostic interview-revised: a revised version of time diagnostic interview for caregivers of individuals with possible pervasive developmental disorders. J Autism Dev Dis.

[46] Marco EJ, Khaibi K, Hill SS, Siegel B, Arroyo MS, Dowling AF (2012). Children with autism show reduced somatosensory response: an MEG study. Autism Res.

[47] Mari M, Castiello U, Marks D, Marraffa C, Prior M (2003). The reach-to-grasp movement in children with autism spectrum disorder. Phil Trans Royal Soc Lon B Bio Sci.

[48] Marrus N, Eggebrecht AT, Todorov A, Elison JT, Wolff JJ, Cole L (2018). Walking, gross motor development, and brain functional connectivity in infants and toddlers. Cereb Cortex.

[49] Ming X, Brimacombe M, Wagner GC (2007). Prevalence of motor impairments in autism spectrum disorders. Brain and Development.

[50] Miyahara M, Tsujii M, Hori M, Nakanshi K, Kageyama H, Sugiyama T (1997). Brief report: motor incoordination in children with Asperger’s syndrome and learning disabilities. J Autism Dev Dis.

[51] Mostofsky SH, Dubey P, Jerath VK, Jansiewicz EM, Goldberg MC, Denckla MB (2006). Developmental dyspraxia is not limited to imitation in children with autism spectrum disorders. J Int Neuropsych Soc.

[52] Mostofsky SH, Ewen JB (2011). Altered connectivity and action model formation in autism is autism. Neuroscientist.

[53] Mugnaini E, Jansen J (1972). The comparative anatomy of histology of the cerebellum: the human cerebellum, cerebellar connections, and cerebellar cortex. Cerebellar cortex, Part II.

[54] Mullen E (1995). Mullen Scales of Early Learning: American Guidance Services.

[55] Mulligan S, Prudhomme White B (2012). Sensory and motor behaviors in infant siblings of children with and without autism. Am J Occup Ther.

[56] Nebel MB, Joel SE, Muschelli J, Barber AD, Caffo BS, Pekar JJ (2012). Disruption of functional organization within the primary motor cortex in children with autism. Hum Brain Mapp.

[57] Noterdaeme M, Mildenberger K, Minow F, Amorosa H (2002). Evaluation of neuromotor deficits in children with autism and children with a specific speech and language disorder. E Child Adol Psych.

[58] Ozonoff S, Iosif A-M, Baguio F, Cook IC, Moore Hill M, Hutman T (2010). A prospective study of the emergence of early behavioral signs of autism. J Am Acad Child Adoles Psych.

[59] Piaget J (1952). The origins of intelligence in the child.

[60] Piek JP, Bradbury GS, Elsley SC, Tate L (2008). Motor coordination and social-communication behavior in preschool-aged children. Int J Disabil Dev Educ.

[61] Provost B, Lopez BR, Heimerl S (2007). A comparison of motor delays in young children: autism spectrum disorder, developmental delay, and developmental concerns. J Autism Dev Dis.

[62] Ritvo ER, Freeman BJ, Scheibel AB, Duong T, Robinson H, Guthrie D (1986). Lower Purkinje cell counts in the cerebella of four autistic subjects: initial findings of the UCLA-NSAC autopsy research report. Am J Psychiatry.

[63] Rochat P (1989). Object manipulation and exploration in 2- to 5-month old infants. Dev Psychol.

[64] Sacrey LR, Armstrong VL, Bryson SE, Zwaigenbaum L (2014). Impairments to visual disengagement in autism spectrum disorder: a review of experimental studies from infancy to childhood. Neurosci Biobeh Reviews.

[65] Sacrey LR, Bryson SE, Zwaigenbaum L (2013). Prospective examination of visual attention during play in infants at high-risk for autism spectrum disorder: a longitudinal study from 6 to 36 months of age. Behav Brain Res.

[66] Sacrey LR, Clark CM, Whishaw IQ (2009). Music attenuates excessive visual guidance of skilled reaching in advanced but not mild Parkinson’s disease. PLoS One.

[67] Sacrey LR, Karl JM, Whishaw IQ (2012). Development of rotational movements, hand shaping, and accuracy in advance and withdrawal for the reach-to-eat movement in human infants aged 6-12 months. Infant Behav Dev.

[68] Sacrey LR, Travis SG, Whishaw IQ (2011). Drug treatment and familiar music aids an attention shift from vision to somatosensation in Parkinson’s disease on the reach- to-eat task. Behav Brain Res.

[69] Sacrey LR, Zwaigenbaum L, Bryson S, Brian J, Smith IM, Roberts W (2015). Can parents’ concerns predict autism spectrum disorder? A prospective study of high-risk siblings from 6 to 36 months of age. J Am Acad Child Adoles Psych.

[70] Schipul SE, Keller TA, Just MA. Inter-regional brain communication and its disturbance in autism. Front Syst Neurosci. 2011;5:10. 10.3389/fnsys.2011.00010. 10.3389/fnsys.2011.00010 PMC3046360 21390284

[71] Shaked M, Yirmiya N (2004). Matching procedures in autism research: evidence from meta-analytic studies. J Autism Dev Disord.

[72] Smith IM, Bryson SE (1998). Gesture imitation in autism I: nonsymbolic postures and sequences. Cog Neuropsychol.

[73] Smyth MM, Anderson HI (2001). Football participation in primary school playground: the role of coordination impairments. Br J Dev Psychol.

[74] Sparrow SS, Balla D, Cicchetti D (1984). Vineland Adaptive Behavior Scales (Survey Form).

[75] Stoit AM, van Schie HT, Slaats-Willemse DI, Buitelaar JK (2013). Grasping motor impairments in autism: not action planning but movement execution is deficient. J Autism Dev Dis.

[76] Teitelbaum O, Benton T, Shah PK, Prince A, Kelly JL, Teitelbaum P (2004). Eshkol-Wachman movement notation in diagnosis: the early detection of Asperger’s syndrome. Proc Natl Acad Sci U S A.

[77] Teitelbaum P, Teitelbaum O, Nye J, Fryman J, Maurer RG (1998). Movement analysis in infancy may be useful for early diagnosis of autism. Proc Natl Acad Sci U S A.

[78] Thach WT (1978). Correlation of neural discharge with pattern and force of muscular activity, joint position, and direction of intended next movement in motor cortex and cerebellum. J Neurophysiol.

[79] Touwen B (1976). Neurological development in infancy. Clinics in developmental medicine, no 58.

[80] Trevarthen C, Delafield-Butt JT (2013). Autism as a developmental disorder in intentional movement and affective engagement. Front Integ Neurosci.

[81] Twitchell TE, Connolly K (1970). Reflex mechanisms and the development of prehension. Mechanisms of motor skill development.

[82] van Swieten LM, van Bergen E, Williams JH, Wilson AD, Plumb MS, Kent SW (2010). A test of motor (not executive) planning in developmental coordination disorder and autism. J Exp Psychol Human Perc Perf.

[83] Volkmar FR, Carter A, Sparrow SS, Cicchetti DV (1993). Quantifying social development in autism. J Am Acad Child Adoles Psych.

[84] Whishaw IQ, Suchowersky O, Davis L, Sarna J, Metz GA, Pellis SM (2002). Impairment of pronation, supination, and body coordination in reach-to-grasp tasks in human Parkinson’s disease (PD) reveals homology to deficits in animal models. Behav Brain Res.

[85] White B, Castle P, Held R (1964). Observations on the development of visually-directed reaching. Child Dev.

[86] Wing L (1981). Asperger’s syndrome: a clinical account. Psych Med.

[87] Yirmiya N, Charman T (2010). The prodrome of autism: early behavioral and biological signs, regression, peri- and post-natal development and genetics. J Child Psychol Psych.

[88] Zwaigenbaum L, Bryson S, Garon N (2013). Early identification of autism spectrum disorders. Behav Brain Res.

[89] Zwaigenbaum L, Bryson S, Rogers T, Roberts W, Brian J, Szatmari P. Behavioral manifestations of autism in the first year of life. Int J Dev Neurosci. 2005;23(2–3):143–52. 10.1016/j.ijdevneu.2004.05.001 15749241

