# Supplementary material for: The reach-to-grasp movement in infants later diagnosed with autism spectrum disorder: a high-risk sibling cohort study
Source: J Neurodev Disord. 2018 Dec 27;10:41. doi: 10.1186/s11689-018-9259-4 (PMC6307213; doi:10.1186/s11689-018-9259-4)
Supplement: Supplementary file 1 — Table S1. Comparison of Main Effects for including all reaches completed between 6 and 36 months versus reaches completed only between 12 and 36 months. (DOCX 22 kb) [file 11689_2018_9259_MOESM1_ESM.docx]

**Additional file 1 for The Reach-to-Grasp Movement in Infants Later Diagnosed with Autism Spectrum Disorder: A High-Risk Sibling Cohort Study**

**Methods**

**Video Recording**

A digital video camera was positioned to record a frontal view of the participant from tabletop to head for video recording at 30 frames/second, with a shutter speed of 500 frames/second (a high shutter speed produces blur-free images and captures rapid eye and hand movements).

**Eshkol-Wachman Movement Notation (EWMN)**

EWMN system has been adapted for the study of human reaching (Foroud & Whishaw, 2010; 2012; Whishaw et al, 2002). In brief, EWMN is designed to express relations and changes in relations between body parts. The body is treated as a system of articulated axes (i.e., body and limb segments). A limb is any body part that either lies between two joints or has a joint and a free extremity. These are imagined as straight lines (axes), of constant length, which move with one end fixed to the centre of a sphere.

An important feature of EWMN is that the same movements can be described in several polar coordinate systems. The coordinates of each system are determined with reference to the environment, to the body midline, and to the next proximal or distal limb or body segment. By transforming the description of the same behavior from one coordinate system to the next, invariances in that behavior may emerge only in some coordinate systems. Thus, the behavior may be invariant in relation to some or all of the following: the participant’s longitudinal axis, gravity, or body-wise in relation to the next proximal or distal segment.

**Original Scoring System**

The movement is divided into seven components:

1. *Orient*: participant moves the head and eyes in order to fixate the target visually prior to reach onset and visually disengage from the target at grasp.
2. *Lift*: hand is lifted and supinated towards the midline of the body as the digits close and semi-flex
3. *Advance*: hand is carried towards the target and stops above the target
4. *Pronation*: hand pronates over target item and digits shape to target size
5. *Grasp*: target is grasped using a pincer grasp (thumb and index) or appropriate
   grasp for object size
6. *Supination*: hand rotates immediately after grasp of target and again as target approaches the mouth
7. *Release*: target is released into the mouth and the hand is returned to its start position

The first five components were used to score our sample, as the present sample did not reliably bring grasped objects to the mouth after ages 12-15 months. As such, only reach-to-grasp scores were included in the analyses.

**Movement Onsets and Offsets**

The onset and offset of the movement phases were identified from the video record. Trial onset was defined as an overt eye movement directed towards the target, or (rarely) the first hand movement towards the target, if occurring before an eye movement towards the target. Trial offset was defined as either (1) an overt eye movement directed away from the target after target grasp, or (2) grasp of the target if the eye disengaged the target before it was grasped. Grasp is defined as the frame before the target was lifted from the substrate, suggesting stable holding of the target (as per Sacrey et al., 2012).

Visual engagements and disengagements were inferred from gaze direction towards and away from the target, respectively. Visual fixation on the target was defined as an overt eye movement directed towards the target, with continuous visual fixation of the target as the hand transported towards the target and the target was grasped. Visual disengagement was defined as an overt eye movement away from the target or a blink accompanied with a redirection of gaze away from the target. Only those visual fixations on the target that were maintained prior to hand movement onset were included in analysis; however, multiple fixations prior to movement onset were rare. Appropriate eye movements were defined as those occurring one second before or after hand movement onset and offset. Sacrey et al. (2012) reported that visual attention in typically developing infants decreased from approximately one second to less than one second for engaging, and from over one second to less than one second for disengaging, between 6 and 12 months of age. Setting the “appropriate” marker for visual attention as one second allows for ‘sticky attention’ to be captured, even in typically developing controls. In addition, any changes to the typical age-related decrease from ‘sticky attention’ to ‘appropriate attention’ in the HR infant siblings will also be detected.

**Results**

Table S1. Comparison of Main Effects for including all reaches completed between 6 and 36 months versus reaches completed only between 12 and 36 months.

| **Component** | **Including reaches from 6 to 36 months** | | | | | | **Including reaches from 12 to 36 months** | | | | | |
| --- | --- | --- | --- | --- | --- | --- | --- | --- | --- | --- | --- | --- |
|  | Group  F p | | Age F p | | Sex F p | | Group  F p | | Age F p | | Sex F p | |
| Orient | 4.08 | .019* | 20.69 | <.001* | 1.97 | .16 | 2.92 | .05* | 12.31 | <.001* | 1.26 | .26 |
| Lift | 6.51 | .002* | 46.60 | <.001* | 3.66 | .06 | 6.75 | .002* | 5.90 | <.001* | 2.52 | .12 |
| Advance | 2.41 | .093 | 67.27 | <.001* | 1.25 | .27 | .89 | .42 | 4.66 | .002* | .06 | .81 |
| Pronation | 6.17 | .003* | 33.14 | <.001* | 10.77 | .001* | 5.96 | .003* | 5.86 | <.001* | 9.93 | .002* |
| Grasp | .61 | .55 | 18.59 | <.001* | 1.39 | .24 | 1.33 | .27 | 2.39 | .05* | 1.62 | .21 |
| R-to-G | 7.66 | .001* | 72.72 | <.001* | 5.41 | .021* | 8.51 | <.001* | 11.61 | <.001* | 6.76 | .011* |

Abbreviations: R-to-G = reach-to-grasp score; * = significant at p<.05
